# Supplementary material for: Hot spot prediction in protein-protein interactions by an ensemble system
Source: BMC Syst Biol. 2018 Dec 31;12(Suppl 9):132. doi: 10.1186/s12918-018-0665-8 (PMC6311905; doi:10.1186/s12918-018-0665-8)
Supplement: Supplementary file 2 — All relevant MATLAB code. A simple Matlab implement of our predictor. (ZIP 19,742 kb) [file 12918_2018_665_MOESM2_ESM.zip › Additional file 2/Read ASA/ASA website/SKEMPI/1BRSD.htm]

 NetSurfP - Protein Surface Accessibility and Secondary Structure Predictions

  

|  |  |  |
| --- | --- | --- |
|  |  | NetSurfP - Protein Surface Accessibility and Secondary Structure Predictions  Technical University of Denmark |

  


---

```
# For publication of results, please cite:
# A generic method for assignment of reliability scores applied to solvent accessibility predictions.
# Bent Petersen, Thomas Nordahl Petersen, Pernille Andersen, Morten Nielsen and Claus Lundegaard
# BMC Structural Biology 2009, 9:51 doi:10.1186/1472-6807-9-51
#
# Column 1: Class assignment - B for buried or E for Exposed - Threshold: 25% exposure, but not based on RSA
# Column 2: Amino acid
# Column 3: Sequence name
# Column 4: Amino acid number
# Column 5: Relative Surface Accessibility - RSA
# Column 6: Absolute Surface Accessibility
# Column 7: Z-fit score for RSA prediction
# Column 8: Probability for Alpha-Helix
# Column 9: Probability for Beta-strand
# Column 10: Probability for Coil
E K  1BRSD                  1    0.792 162.997   1.508   0.005   0.015   0.979
E K  1BRSD                  2    0.513 105.483   1.730   0.001   0.900   0.099
B A  1BRSD                  3    0.097  10.667   0.489   0.001   0.959   0.040
E V  1BRSD                  4    0.384  59.005   1.104   0.001   0.959   0.040
B I  1BRSD                  5    0.080  14.782   0.237   0.001   0.959   0.040
E N  1BRSD                  6    0.388  56.818   0.410   0.002   0.816   0.182
B G  1BRSD                  7    0.170  13.348  -1.381   0.020   0.205   0.775
E E  1BRSD                  8    0.569  99.474   0.040   0.018   0.088   0.893
E Q  1BRSD                  9    0.524  93.515   1.338   0.021   0.279   0.699
B I  1BRSD                 10    0.069  12.673   0.188   0.021   0.279   0.699
E R  1BRSD                 11    0.510 116.813   0.538   0.019   0.141   0.840
E S  1BRSD                 12    0.442  51.826   0.590   0.018   0.019   0.964
E I  1BRSD                 13    0.295  54.538   1.215   0.923   0.002   0.076
E S  1BRSD                 14    0.676  79.192   0.837   0.970   0.001   0.030
E D  1BRSD                 15    0.468  67.453   1.867   0.970   0.001   0.030
B L  1BRSD                 16    0.049   8.899   0.860   0.970   0.001   0.030
B H  1BRSD                 17    0.162  29.395   1.115   0.970   0.001   0.030
E Q  1BRSD                 18    0.584 104.213   1.504   0.970   0.001   0.030
E T  1BRSD                 19    0.314  43.566   2.094   0.988   0.000   0.012
B L  1BRSD                 20    0.042   7.709   0.542   0.970   0.001   0.030
E K  1BRSD                 21    0.268  55.169   1.315   0.970   0.001   0.030
E K  1BRSD                 22    0.687 141.295   1.194   0.923   0.002   0.076
E E  1BRSD                 23    0.402  70.264   1.105   0.858   0.002   0.139
B L  1BRSD                 24    0.100  18.402   0.506   0.406   0.004   0.590
E A  1BRSD                 25    0.733  80.777   0.548   0.016   0.005   0.979
B L  1BRSD                 26    0.157  28.747   0.144   0.003   0.003   0.994
E P  1BRSD                 27    0.578  81.976  -0.172   0.003   0.003   0.994
E E  1BRSD                 28    0.712 124.439  -0.121   0.016   0.005   0.979
B Y  1BRSD                 29    0.233  49.835   0.020   0.181   0.016   0.803
B Y  1BRSD                 30    0.235  50.241  -0.014   0.339   0.016   0.645
B G  1BRSD                 31    0.277  21.816  -1.288   0.257   0.016   0.727
E E  1BRSD                 32    0.434  75.785   0.219   0.257   0.016   0.727
B N  1BRSD                 33    0.204  29.866  -0.663   0.181   0.016   0.803
B L  1BRSD                 34    0.079  14.520   1.084   0.522   0.016   0.462
E D  1BRSD                 35    0.253  36.501  -0.200   0.717   0.014   0.269
B A  1BRSD                 36    0.079   8.750   0.187   0.879   0.010   0.111
B L  1BRSD                 37    0.032   5.786  -0.407   0.879   0.010   0.111
B W  1BRSD                 38    0.113  27.297   0.488   0.879   0.010   0.111
B D  1BRSD                 39    0.186  26.817  -0.450   0.879   0.010   0.111
B A  1BRSD                 40    0.049   5.411   0.413   0.879   0.010   0.111
B L  1BRSD                 41    0.052   9.448   0.374   0.717   0.014   0.269
B T  1BRSD                 42    0.269  37.310   0.047   0.430   0.016   0.555
E G  1BRSD                 43    0.510  40.121  -1.498   0.115   0.016   0.868
E W  1BRSD                 44    0.381  91.606  -1.220   0.058   0.017   0.925
B V  1BRSD                 45    0.111  17.061  -0.328   0.053   0.043   0.903
E E  1BRSD                 46    0.613 107.144  -0.304   0.053   0.043   0.903
B Y  1BRSD                 47    0.232  49.493   1.122   0.018   0.047   0.935
B P  1BRSD                 48    0.199  28.196  -0.270   0.019   0.141   0.840
B L  1BRSD                 49    0.197  36.107   0.625   0.021   0.756   0.223
E V  1BRSD                 50    0.380  58.375   1.074   0.011   0.918   0.071
B L  1BRSD                 51    0.060  11.059   1.056   0.006   0.962   0.032
E E  1BRSD                 52    0.287  50.174   1.423   0.001   0.959   0.040
B W  1BRSD                 53    0.068  16.330   0.752   0.001   0.959   0.040
E R  1BRSD                 54    0.420  96.111   1.722   0.003   0.718   0.279
E Q  1BRSD                 55    0.369  65.850  -1.030   0.005   0.262   0.733
B F  1BRSD                 56    0.174  34.982   0.186   0.268   0.043   0.689
E E  1BRSD                 57    0.629 109.851   0.910   0.694   0.003   0.303
E Q  1BRSD                 58    0.497  88.728   1.243   0.782   0.003   0.216
B S  1BRSD                 59    0.095  11.134  -0.700   0.694   0.003   0.303
E K  1BRSD                 60    0.321  65.947   0.369   0.600   0.003   0.397
E Q  1BRSD                 61    0.693 123.788   0.966   0.600   0.003   0.397
E L  1BRSD                 62    0.392  71.739   0.570   0.502   0.002   0.495
B T  1BRSD                 63    0.138  19.210  -0.404   0.246   0.004   0.750
E G  1BRSD                 64    0.443  34.888  -0.312   0.176   0.004   0.820
E A  1BRSD                 65    0.329  36.256   0.510   0.782   0.003   0.216
E E  1BRSD                 66    0.554  96.749   0.741   0.923   0.002   0.076
E S  1BRSD                 67    0.287  33.636   1.575   0.970   0.001   0.030
B V  1BRSD                 68    0.036   5.579   0.246   0.970   0.001   0.030
E L  1BRSD                 69    0.222  40.630   1.932   0.970   0.001   0.030
E Q  1BRSD                 70    0.528  94.372   1.929   0.970   0.001   0.030
B V  1BRSD                 71    0.052   7.977   1.349   0.970   0.001   0.030
B F  1BRSD                 72    0.051  10.155   0.975   0.970   0.001   0.030
E R  1BRSD                 73    0.478 109.554   1.866   0.970   0.001   0.030
E E  1BRSD                 74    0.414  72.343   1.801   0.970   0.001   0.030
B A  1BRSD                 75    0.029   3.196   0.534   0.970   0.001   0.030
E K  1BRSD                 76    0.291  59.838   0.859   0.923   0.002   0.076
E A  1BRSD                 77    0.691  76.170   0.888   0.858   0.002   0.139
E E  1BRSD                 78    0.465  81.288   0.241   0.782   0.003   0.216
B G  1BRSD                 79    0.262  20.604   0.445   0.321   0.003   0.675
E A  1BRSD                 80    0.727  80.082   0.177   0.016   0.005   0.979
E D  1BRSD                 81    0.767 110.568   0.959   0.005   0.015   0.979
B I  1BRSD                 82    0.076  14.060   0.812   0.005   0.336   0.660
E T  1BRSD                 83    0.486  67.394   1.668   0.002   0.816   0.182
B I  1BRSD                 84    0.124  22.847   0.558   0.011   0.918   0.071
B I  1BRSD                 85    0.203  37.555  -0.065   0.021   0.756   0.223
B L  1BRSD                 86    0.256  46.800  -0.142   0.022   0.552   0.426
E S  1BRSD                 87    0.806  94.416   0.293   0.003   0.003   0.994

Explain the output.  Go back.
```

---
